# Beyond cellulose: pharmaceutical potential for bioactive plant polysaccharides in treating disease and gut dysbiosis

**DOI:** 10.3389/fmicb.2023.1183130

**Published:** 2023-05-24

**Authors:** Yuanlin Niu, Wei Liu, Xueni Fan, Dongxu Wen, Dan Wu, Hongzhuang Wang, Zhenjiang Liu, Bin Li

**Affiliations:** ^1^Key Laboratory of Animal Genetics and Breeding on Tibetan Plateau, Ministry of Agriculture and Rural Affairs, Institute of Animal Husbandry and Veterinary, Tibet Academy of Agricultural and Animal Husbandry Sciences, Lhasa, China; ^2^School of Public Health, Lanzhou University, Lanzhou, China; ^3^National Engineering Laboratory for AIDS Vaccine, School of Life Sciences, Jilin University, Changchun, China

**Keywords:** polysaccharides, gut microbiota, structure, bioactivities, prebiotics

## Abstract

Polysaccharides derived from plants, algae, or fungi serve as the major components of some human diets. Polysaccharides have been shown to exhibit diverse biological activities in improving human health, and have also been proposed to function as potent modulators of gut microbiota composition, thus playing a bi-directional regulatory role in host health. Here, we review a variety of polysaccharide structures potentially linked to biological functions, and cover current research progress in characterizing their pharmaceutical effects in various disease models, including antioxidant, anticoagulant, anti-inflammatory, immunomodulatory, hypoglycemic, and antimicrobial activities. We also highlight the effects of polysaccharides on modulating gut microbiota *via* enrichment for beneficial taxa and suppression of potential pathogens, leading to increased microbial expression of carbohydrate-active enzymes and enhanced short chain fatty acid production. This review also discusses polysaccharide-mediated improvements in gut function by influencing interleukin and hormone secretion in host intestinal epithelial cells.

## 1. Introduction

**Figure 1:**
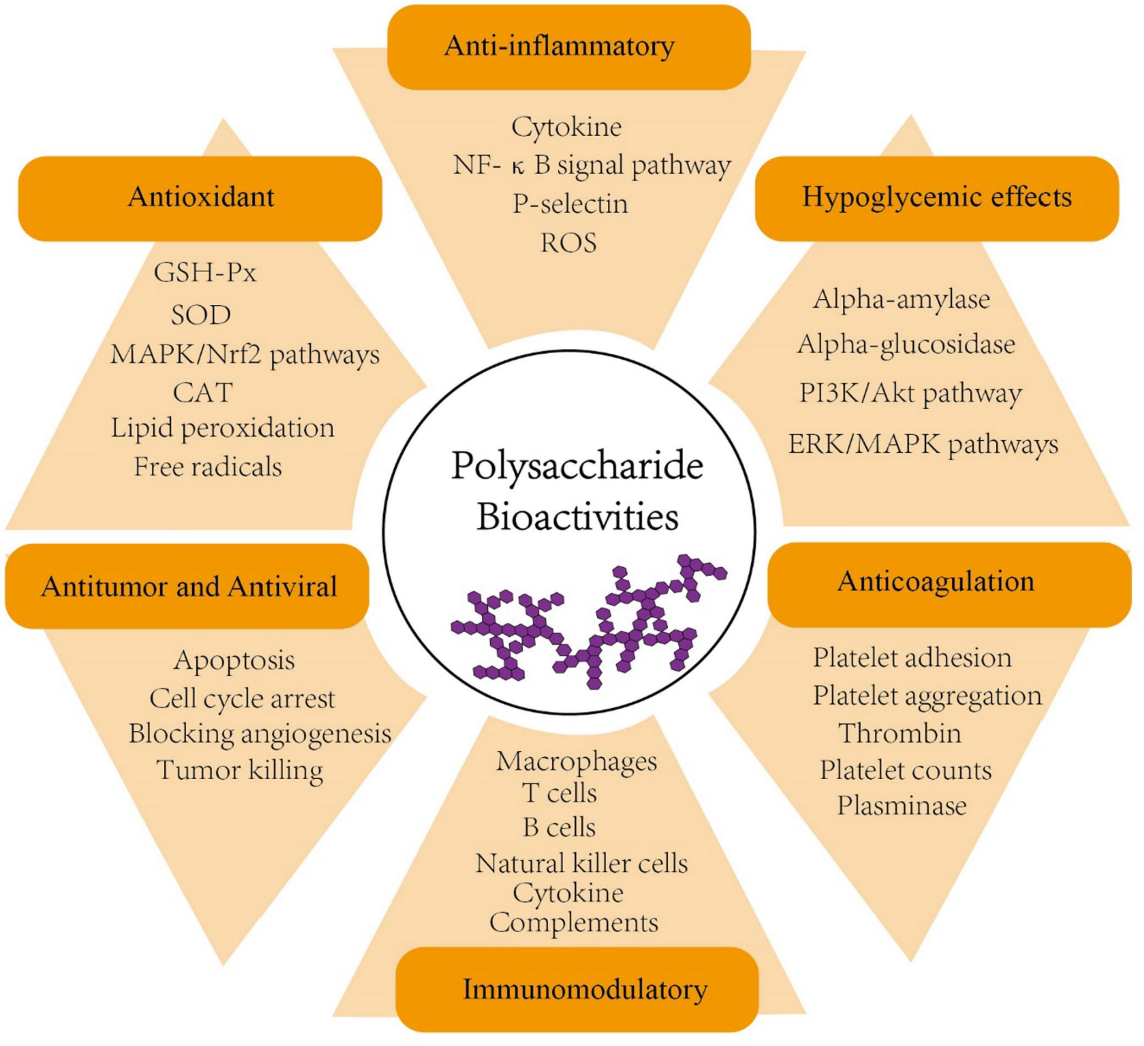
Bioactivities of polysaccharides (anticoagulation, antiviral, antitumor, antioxidant, hypoglycemic, anti-inflammatory and immunomodulatory). PI3K, phosphoinositide 3-kinase; AKT, serine/threonine-specific protein kinase; ERK, extracellular-signal-regulated kinase; MAPK, mitogen-activated protein kinase; NF-κB, nuclear factor-κB; ROS, reactive oxygen species; GSH-Px, glutathione peroxidase; SOD, superoxide dismutase; CAT, catalase; Nrf2, nuclear factor erythroid 2–related factor 2.

Polysaccharides, formed by *α*- or *β*-glycosidic bond of identical or various monosaccharide monomers with10 or more polymerization, are naturally produced in large quantities by plants and fungi (Tan et al., 2017). To date, numerous polysaccharides have been shown to exhibit a range of biological activities, including anticoagulation, antiviral, antitumor, antioxidant, hypoglycemic, and immunomodulatory effects (Zeng et al., 2019; Zhang et al., 2019; Wang et al., 2019b; Kalinina et al., 2020; Chaisuwan et al., 2021; Chen C. et al., 2021; Kiddane and Kim, 2021; Liang Q. et al., 2021; Surayot et al., 2021; Figure 1 and Table 1). In addition, many studies have proposed that some polysaccharides also contribute to shaping the structure, diversity and function of gut microbiota and thus play a role in enhancing human health (Koropatkin et al., 2012; Chang et al., 2015; Lin et al., 2018; Zhang L. et al., 2018; Zhang X. et al., 2018; Chen G. et al., 2019; Ding et al., 2019; Wang M. et al., 2019; Wang Y. et al., 2020; Wang et al., 2020b; Guo et al., 2021a).

**Table 1:** The action of some bioactive polysaccharides in disease prevention and treatment.

Source	Polysaccharide	Disease	Action	References
*Laminaria japonica*	Fucoidan	Parkinson’s disease (PD)	Reversed the loss of nigral dopaminergic neurons and striatal dopaminergic fibers	Zhang L. et al. (2018) and Zhang X. et al. (2018)
*Inonotus obliquus*	Polysaccharide	Alzheimer’s disease (AD)	Improved the pathological behaviors correlated with memory and cognition, upregulated Nrf2 expression and its downstream proteins, decreased *β*-amyloid peptides deposition and neuronal fiber tangles	Han et al. (2019)
Walnut green husk	Polysaccharide	Inflammatory bowel disease (IBD)	Enhanced the Production of SCFAs through fermentation in the colon which help on allivating inflammatory damage and protecting integrity of the intestinal barrier function	Wang et al. (2021)
Walnut green husk	Polysaccharide	Obesity	Relieved the oxidative stress in the liver by modulating the MAPK/Nrf2 pathway, and promoted the browning of inguinal white adipose tissue and thermogenesis in brown adipose tissue.	Wang et al. (2021)
*Hypsizygus ulmarius*	Polysaccharide	Diabetes mellitus (DM)	Exhibited moderate inhibition activity against *α*-amylase and *α*-glucosidase enzyme in a concentration-dependent manner	Govindan et al. (2023)
*Momordica charantia* L	Selenylated polysaccharide (Se-MCPIIa-1)	DM	Reduced fasting blood glucose levels and increased insulin levels	Ru et al. (2020)
Hawthorn (*Crataegus*.)	Polysaccharide	Colon cancer	Arrested the cell cycle in the S and G2/M phases, increased the rate of apoptosis, downregulated the expression of Cyclin A1/D1/E1 and CDK-1/2	Ma et al. (2020)
*Crataegus pinnatifida*	Polysaccharide	Colitis	Restored the pathological lesions in colon, decreased the expression of inflammatory cytokines (IL-1β, IL-6, and TNF-α)	Guo et al. (2021b)
*Lactobacillus plantarum* YW11	Exopolysaccharide	IBD	Inhibited inflammatory cytokines (TNF-α, IL-1β, IL-6, IFN-γ and IL-12) and enhanced the anti-inflammatory cytokine IL-10.	Min et al. (2020)
*Bacillus subtilis*	Exopolysaccharide	Bacterial infections	Limited superantigens-T cell activation by *S. aureus* and abrogated systemic induction of gamma interferon.	Paik et al. (2019)
Red seaweed *Gelidium pacificum* Okamura	Sulfated polysaccharide	Antibiotic-associated diarrhea (AAD)	Promoted the recovery of the gut microbiota and improved mucosal barrier function, downregulated the levels of inflammatory cytokines and enhanced the production of SCFAs.	Cui et al. (2020)

*Nrf2, nuclear factor erythroid 2–related factor 2; SCFA, short-chain fatty acids; MAPK, mitogen-activated protein kinase; CDK1/2, cyclin dependent kinase-1/2; IL, interleukin; IFN, interferon; TNF, tumor necrosis factor.

Gut microbes share a symbiotic lifestyle with human hosts, colonizing the gastrointestinal tract, and a large number of studies have highlighted the significance of these microbes, collectively termed microbiota, in mediating interactions that determine human health or disease states involving the gastrointestinal, cardiovascular, orthopedic and even neurological systems (Björkstén et al., 2001; Li et al., 2019). Recently, naturally occurring undigestable polysaccharides in plants and foods were found to exert regulatory effects on gut microbiota by selecting for beneficial microorganisms in the gut while inhibiting the growth of pathogenic bacteria. This activity reinforces the structural and functional integrity of the intestinal mucosal barrier, and enhances the intestinal immune system *via* modulation of cytokine expression levels (de Vrese and Schrezenmeir, 2008; Chiu et al., 2014; Fuke et al., 2019; Han et al., 2020; Zhang et al., 2020; Zhao et al., 2020; Guo et al., 2021b). For example, Hawthorn HAW1-2 Polysaccharide (Guo et al., 2021b), *Ziziphus Jujuba* Polysaccharide (Han et al., 2020), glycyrrhiza polysaccharide (Zhang X. et al., 2018), and *Lycium barbarum* polysaccharide (Ding et al., 2019) were all reported to act as prebiotics by affecting gut microbiota structure and diversity. In addition, fungal polysaccharides were also found to play an important regulatory role as prebiotics through mechanisms similar to that of plant polysaccharides (Liang J. et al., 2021). However, studies of these various prebiotic effects of polysaccharides have yet to fully uncover the full range of interaction mechanisms between polysaccharides and gut microbiota. Thus, a summary of the different plant sources and structures of known bioactive polysaccharides can facilitate ongoing research efforts, especially their prebiotic effects and modulation of microbiota in the context of human health. It should be noted that progress toward understanding the diversity of prebiotic polysaccharide functions requires the integration of plant phenotypic data with multi-omics analyses to identify tripartite host-polysaccharide-microbiota interactions.

## 2. Overview of polysaccharides and their bioactivities

### 2.1. Structure and classification of polysaccharides

**Figure 2:**
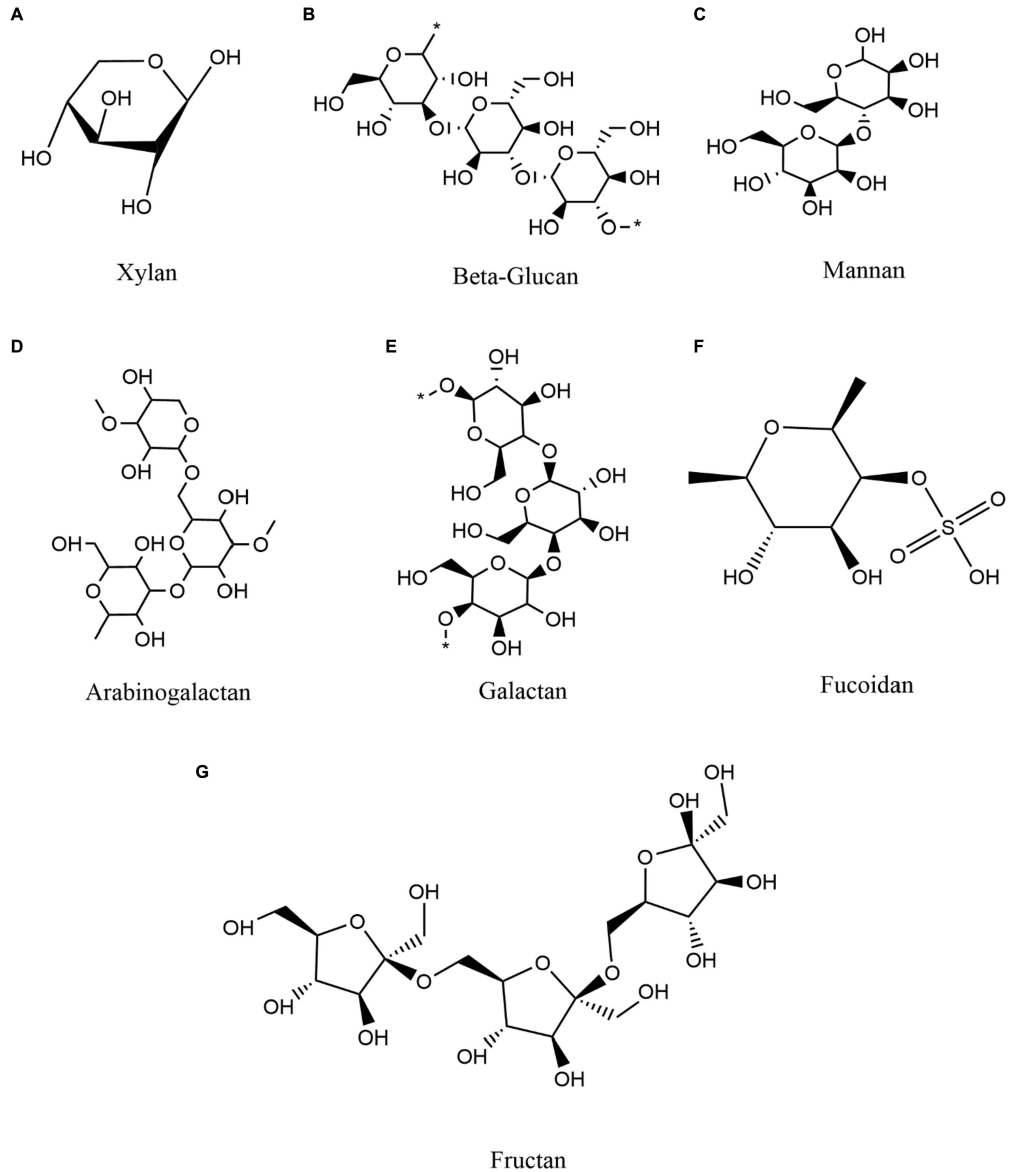
Classification and composition of some representative polysaccharides and commonly polymerized monosaccharides. **(A)** Xylan **(B)** Beta-Glucan **(C)** Mannan **(D)** Arabinogalactan **(E)** Galactan **(F)** Fucoidan **(G)** Fructan.

To provide an overview of higher order polysaccharide structures, polysaccharides are first categorized by their primary structure, determined by connection types, the organization and composition of sugar residues, the configuration of glycosidic bonds, and the conformation of sugar rings (Yang et al., 2020). The secondary structure of oligosaccharides, i.e., their regular conformation resulting from hydrogen bonding (the most common secondary bonds between main chains), is determined by the dihedral Φ, Ψ or ω angles of the polymer backbone (Hatcher et al., 2011). Based on the secondary structure of polysaccharides, the tertiary structure is formed by non-covalent interactions among carboxyl groups, hydroxyl groups, sulfate groups, and/or amino groups on the sugar units. The polysaccharide quaternary structure refers to aggregates formed by non-covalent bonding between polymer chains (Lafond et al., 2016). In addition, polysaccharides can be classified as either homopolysaccharides or heteropolysaccharides, homo-polysaccharides are composed of a single type of monosaccharide and hetero-polysaccharides are composed of different types of monosaccharides (Sinha and Kumria, 2001; Liu et al., 2008). For example, glucans are glucose homopolysaccharides, while mannans are mannose homopolysaccharides (d’Ayala et al., 2008). Overall, polysaccharides are categorized as glucans, mannans, pectin polysaccharides, arabinogalactans, galactans, fucoidan, fructan, and polyxylose, among others, based on their monosaccharide composition and linkage types (Tan et al., 2017; Maji, 2019; Figure 2).

Different monosaccharide contents and branching exhibit various bioactivities. Wu et al. (2020) isolated polysaccharide from the seeds of *Litchi chinensis Sonn*. and reported reduction of antitumor activity of branched backbone of polysaccharides with more than four monosaccharide units. The monosaccharide contents of polysaccharides could also influence their anti-inflammatory activity (Chen Y. C. et al., 2019). In addition, polysaccharides contain many functional groups, including hydroxyl groups and a hemiacetal reducing end that has the potential to reduce precursor salts (Park et al., 2011). Studies have revealed that the oxidation of polysaccharide hydroxyl groups to carbonyl groups play a significant role in reducing gold salts (Mata et al., 2009). Alternatively, the reducing end of polysaccharides has been used to introduce an amino functional group capable of forming complexes with and stabilizing metallic nanoparticles (Nadkarni et al., 1994). Carbohydrates with these amino groups bind strongly to the surface of gold or silver nanoparticles to provide a hydrophilic surface (Kemp et al., 2009). The introduction of specific functional groups will change a polysaccharide’s molecular weight, structure, types, position and number of substituents groups, and may alter the physiochemical and functional properties of that polysaccharide (Li et al., 2016). More specifically, various modifications, including sulfation, phosphorylation, carbomethylation, benzoylation or acetylation have all been shown to enhance the biology activity of some specific polysaccharides (Xu Y. et al., 2019). For instance, in heparan sulfate (HS) polysaccharides, negatively charged sulfate and carboxylate decorations can be organized into a variety of different so-called HS S-domains through a tightly controlled biosynthetic pathway that enables remarkable structural variability (Li and Kusche-Gullberg, 2016). Using standard DPPH and ABTS assays, Cao et al. (2020) characterized the antioxidant properties of sulfated polysaccharides from *Amana edulis* (Cao et al., 2020). Another study illustrated the positive effects of phosphorylation- based modifications on increasing the antioxidant activity of polysaccharides isolated from *Momordica charantia* (Chen F. et al., 2019). Recently, nanocellulose incorporated polysaccharides were reported to extent nanoparticle application in health promotion. Incorporation of chitosan and nanocellulose could improve their antimicrobial activity (Tomé et al., 2013), as well as exerting roles in wound healing (Hasan et al., 2017). These different combinations of functional groups of polysaccharides from diverse sources, along with monosaccharide composition, linkage types, and chemical modifications can thus result in different biological activities.

### 2.2. Polysaccharide bioactivities

#### 2.2.1. Anticoagulation and anti-inflammatory effects

Polysaccharides are widely reported to exhibit anticoagulation properties by inhibiting thrombin activity, decreasing platelet counts, inhibiting platelet adhesion and aggregation, enhancing plasminase activity, and promoting the dissolution of fibrin (Matsubara et al., 2001; Pawlaczyk et al., 2011; Xue et al., 2018). Souza et al. (2015) identified polysaccharides from spinosa bark that could provide anticoagulant, antiplatelet and antithrombotic effects without increasing inducing a greater likelihood of hemorrhage (Souza et al., 2015). Similarly, an acidic polysaccharide was extracted from the edible mushroom *Auricularia auricula* which contained mannose, glucose, glucuronic acid and xylose oligosaccharides, but no sulfate ester links. This polysaccharide displayed potent anticoagulation effect by inhibiting thrombin *via* antithrombin activation (Yoon et al., 2003). Sulfated galactans produced by the seaweed *Hypnea musciformis* were also found to have antioxidant, anticoagulant, and immunostimulatory properties, depending on the method of their extraction (Gabriela das Chagas Faustino Alves et al., 2016).

Other than anticoagulant effects, polysaccharides are also known to exert anti-inflammatory activities. Fu et al. (2022) studied the structure of polysaccharides from Chinese aconite (*Aconitum carmichaelii*) leaves and demonstrated that these polysaccharides have immunomodulatory and anti-inflammatory effects on lipopolysaccharide (LPS)-induced inflammation in intestinal epithelial cells (Fu et al., 2022). In addition, *in vitro* investigation of the bioactivity of polysaccharides from *Typha angustifolia* using RAW264.7 cell provided evidence indicating that these polysaccharides can significantly suppress inflammatory cytokine production, nuclear factor-κB (NF-κB) signal pathway activation, and reactive oxygen species (ROS) production (Wei et al., 2021). Study on *Sargassum fusiforme* fucoidan has shown that polysaccharides can inhibit selectin-mediated leukocyte migration and infiltration by blocking interactions between P-selectin and its ligands on leukocytes, ultimately reducing the levels of IL-6, IL-8, TNF, and CRP cytokines to ameliorate systemic inflammation (Wu S. et al., 2019). Guo et al. (2018) studied the anti-inflammatory activity and related mechanism of polysaccharides isolated from *Sargentodoxa cuneata.* Their findings demonstrated that these polysaccharides could markedly inhibit carrageenan-induced edema in the hind paw of rats by decreasing malondialdehyde and prostaglandin E2 levels in the hind paw, serum and liver, while promoting SOD activity in serum and liver (Guo et al., 2018).

#### 2.2.2. Immunomodulatory effects

Numerous polysaccharides from fungi and plants can reportedly provide various dietary and medicinal benefits, including marked effects on immune system function. Polysaccharides have also been shown to function as immunomodulators through a variety of mechanisms (Mousavi et al., 2015), such as activating macrophages, T cells, B lymphocytes, or natural killer cells, or by activating complements and promoting cytokine production (Kouakou et al., 2013). This regulation of innate immune response can substantially impact the host’s ability to rapidly respond to pathogens. As an essential component of the host immune system, macrophages collaborate with other cell types, such as neutrophils, to resist the adverse effects of biotic and abiotic stresses (Schepetkin and Quinn, 2006; Shen et al., 2014). To augment this function, some polysaccharide signal molecules can activate a macrophage-mediated immune response *via* binding with different receptors on macrophages, such as Toll-like receptor 4, complement receptor 3, scavenger receptor, mannose receptor, and Dectin-1, consequently initiating one or more intracellular signaling cascades that ultimately result in production of inflammation-related cytokines (Schepetkin and Quinn, 2006; Liao et al., 2015; Bunyatyan et al., 2017; Gong et al., 2017; Zhou et al., 2020). These macrophage-associated immunomodulatory effects of plant polysaccharides are largely mediated by increased ROS generation, cytokine secretion, cell proliferation, and phagocytic activity of macrophages (Gong et al., 2017) One earlier study described a novel polysacchaide obtained from the fruiting body of *Dictyophora indusiate* that could significantly promote macrophage secretion of NO, TNF-α, and IL-6 *via* complement receptor 3 in mouse RAW 264.7 cells (Liao et al., 2015). As organic selenium compound with complex chemical structure and diverse sources, selenium polysaccharide has been widely studied as its biological activities. A wide variety of non-specific immune cells, such as natural killer cells and macrophages display significantly improved immune function in the presence of selenium polysaccharides (Zhou et al., 2020). These advances in understanding the scope of polysaccharide activity suggest that many more plant polysaccharides with immunomodulatory effects have yet to be identified through extensive screening and research.

#### 2.2.3. Hypoglycemic effects

Diabetes is a chronic, metabolic disease with typical hyperglycemia symptoms which is characterized by insulin resistance and a relative/absolute insulin insufficiency (Bunyatyan et al., 2017). Plant polysaccharides can also stimulate insulin secretion, modulate the activity of glucose metabolizing enzymes, inhibit the gluconeogenesis pathway, and promote glucose utilization in peripheral tissues, thus performing important functions in the prevention and treatment of diabetes (Wu et al., 2016). Tea made with guava (*Psidium guajava* L., Myrtaceae) leaves has long been used as a traditional herbal treatment for diabetes in Asia and North America (Oh et al., 2005). Polysaccharides from guava leaf have been shown to exhibit potent free-radical scavenging activity toward DPPH, OH, and ABTS, and can significantly lower fasting blood sugar, total cholesterol, total triglyceride, glycated serum protein, creatinine, and malonaldehyde levels (Luo et al., 2019). In addition, treatment with these polysaccharides can significantly increase total antioxidant activity and superoxide dismutase (SOD) enzyme activity in diabetic mice, consequently ameliorating damage to the liver, kidney, and pancreas (Luo et al., 2019). Recently, the application of bitter gourd (*Momordica charantia*) as herbal medicine/vegetable in the treatment against diabetes has been widely reported. One study investigating the hypoglycemic effects of *Momordica charantia* polysaccharides in alloxan-induced diabetic mice model showed that the polysaccharide treatment led to significantly lower fasting blood glucose levels and improved glucose tolerance, thus proposed dose-dependent anti-diabetic activity (Xu et al., 2015). In order to obtain better antidiabetic activities, a selenylated polysaccharide from *Momordica charantia* has been applied to diabetic mice and was reported to prevent pancreatic islets, liver and kidney damage from diabetes by reduction of fasting blood glucose levels, enhancement of insulin levels and antioxidant enzyme activities (Ru et al., 2020). The pumpkin polysaccharides also demonstrated a significant glucose tolerance effect, and effectively alleviated insulin resistance in addition to providing cytoprotective benefits on type II diabetes mellitus (T2DM) mice (Chen X. et al., 2019). In particular, glucomannan and glucogalactan have been shown to exhibit significant antidiabetic properties by inhibiting alpha-amylase and alpha-glucosidase activity to promote pancreatic beta cell proliferation and stimulate insulin sensitivity and secretion (Mirzadeh et al., 2021).

**Figure 3:**
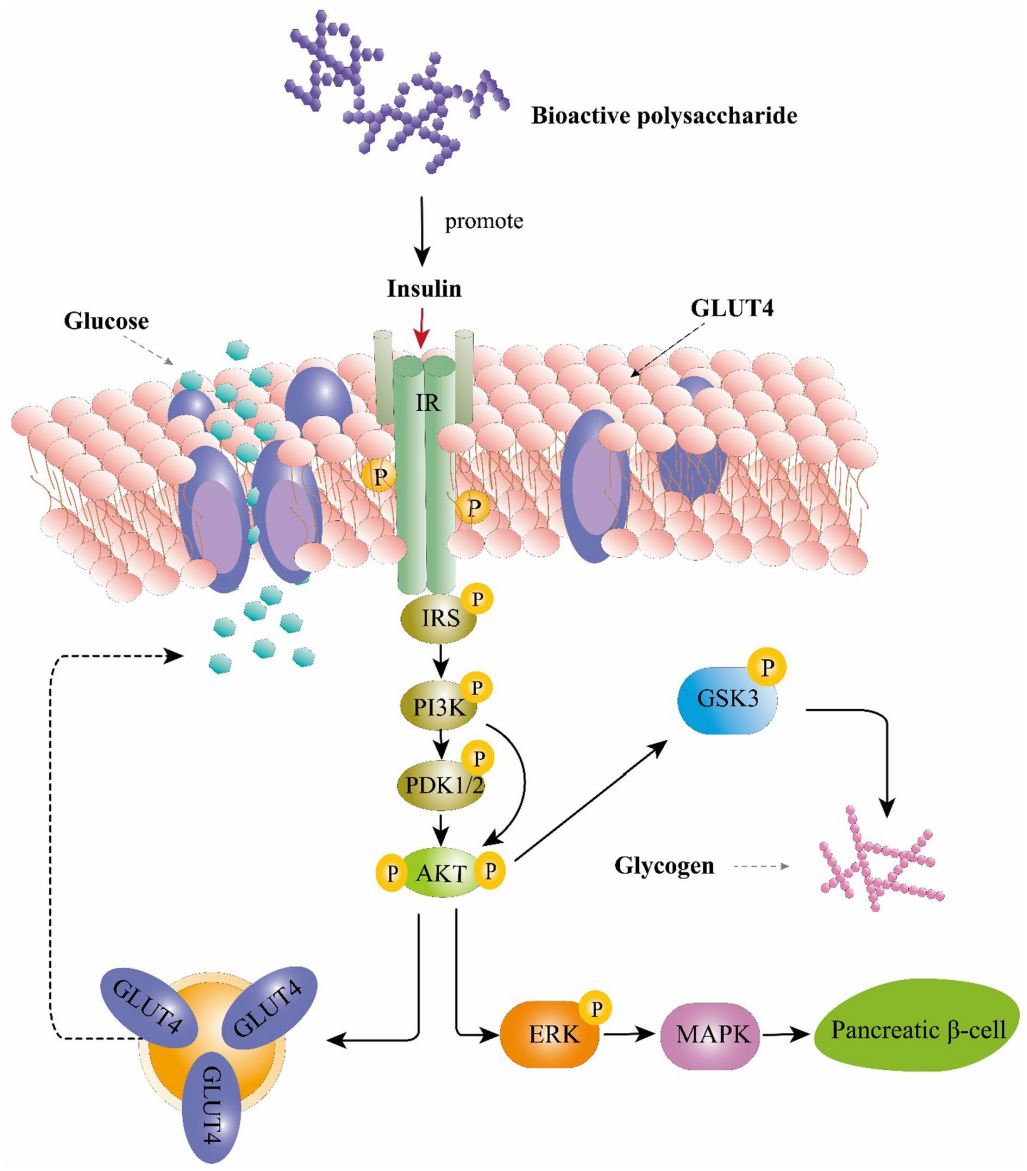
The hypoglycemic function of bioactive polysaccharides on the insulin signaling pathway. IRS, insulin receptor substrate; PI3K, phosphoinositide 3-kinase; PDK1/2, phosphoinositide-dependent protein kinase 1 and 2; AKT, serine/threonine-specific protein kinase; GSK-3, glycogen synthase kinase-3; GLUT4, glucose transporter type 4; ERK, extracellular-signal-regulated kinase; MAPK, mitogen-activated protein kinase.

Mechanistically, these effects are mediated by phosphorylated tyrosine residues present in the intracellular substrates of the insulin receptor (IRS) (Mirzadeh et al., 2021). These substrates could activate the phosphoinositide 3-kinase (PI3K), phosphoinositide-dependent protein kinase 1 and 2 (PDK1/2) and then activates serine/threonine-specific protein kinase (AKT) pathways (Ganesan and Xu, 2019). AKT activates the phosphorylation of glycogen synthase kinase 3 (GSK3), resulting in upregulation of glycogen synthesis in the liver and skeletal muscle (Mirzadeh et al., 2021). Moreover, AKT can also stimulate the translocation of glucose transporter 4 (GLUT4) to the plasma membrane which consequently enhance glucose uptake (Wang et al., 2012). Study also found that AkT is a main mediator in activating the extracellular-signal-regulated kinase (ERK) /mitogen-activated protein kinase (MAPK) pathways thereby triggering several physiological and biochemical mechanisms, such as cell differentiation, proliferation, apoptosis, and cell endurance (Jayachandran et al., 2019). IRS studies on *Ophiopogon japonicus* polysaccharide and glucopyranose-rich heteropolysaccharide from *Catathelasma ventricosum* demonstrated that these compounds could trigger the PI3K/AKT signaling pathway through IRS1, PI3K-p85, and phosphorylated AKT to promote insulin sensitivity and improve diabetes-associated renal disease (Wang et al., 2012; Liu et al., 2016a,b). Collectively, polysaccharide could regulate glucose uptaking, glycogen synthesis and β-cell activity through PI3K/Akt pathway and ERK/MAPK pathways and resulting in playing an anti-diabetic role (Figure 3).

#### 2.2.4. Antioxidant effects

Oxidative stress can be link to a variety of diseases including cancer, cardiovascular diseases, diabetes, respiratory diseases, immune deficiency and neurodegenerative disorders, while antioxidants could protect cells against free radicals and reduce the risk of many diseases (Fridovich, 1999; Fang et al., 2002). Plant polysaccharides have been shown to directly eliminate free radicals by inhibiting lipid peroxidation, scavenging hydroxyl free radicals, and clearing superoxide anion free radicals. They also act on free radicals indirectly by enhancing the activities of SOD, catalase (CAT), and glutathione peroxidase to maintain a balance of free radicals, which can collectively diminish or avert the occurrence of disease (Xie et al., 2016). Lin et al. (2022) used enzymatic and microwave extraction methods to obtain polysaccharides from Purple-Heart Radish (*Raphanus sativus*) that displayed high antioxidant effects by inhibiting lipid peroxidation (Lin et al., 2022). Studies of polysaccharides from yerba mate (*Ilex paraguariensis*) tea reported IC50 values of 3.36 ± 0.31 mg/mL for ·OH scavenging activity, suggesting a strong antioxidant capacity (Chen X. et al., 2019). Xu et al. (2012) characterized polysaccharides from flowers of *Camellia sinensis* and found a high, dose-dependent capacity for scavenging superoxide anion free radicals (Xu et al., 2012). Study of polysaccharides from *Astragalus membranaceus* (Fisch.) has shown that ROS levels decrease, SOD activity increases, and superoxide dismutase free radical scavenging is enhanced, which can alleviate tissue damage and delay senescence (Song et al., 2019). Shan et al. (2011) found that administering *Lycium barbarum* polysaccharides led to significantly higher SOD and glutathione peroxidase (GPX) levels in rats with exercise-induced oxidative stress, indicating that these polysaccharides played a significant role in preventing oxidative stress after exhaustive exercise (Shan et al., 2011). Investigation of alfalfa polysaccharides illustrated their antioxidant effects on immune response in preventing H_2_O_2_-induced oxidative damage by activating mitogen-activated protein kinase (MAPK)/nuclear factor erythroid 2–related factor 2 (Nrf2) signaling pathways while suppressing NF-κB signaling in mouse embryonic fibroblasts (Wang et al., 2019a).

In addition to naturally occurring polysaccharides, chemical modification can also enhance the antioxidant effects of some polysaccharides. For example, findings by Gao et al. (2017) showed that selenylation of *Angelica sinensis* polysaccharide could enhance its antioxidant and hepatoprotective activity through inhibition of p- ERK and p-JNK signaling in mice with hepatic injury (Gao et al., 2017). Collectively, polysaccharides from diverse sources have been applied as functional antioxidant components in many pharmaceutical/nutraceutical products due to their capacity for modulating ROS levels, enzymatic and non-enzymatic antioxidant defense responses (e.g., SOD, CAT, GPX), and oxidative stress-induced signaling pathways (e.g., MAPK ERK, and JNK).

#### 2.2.5. Antitumor and antiviral capacities

Cancer remains one of the greatest threats to public health worldwide, and is a long-standing focus of research attention and drug development, especially those with reduced side-effects. Bioactive polysaccharides have been identified that exert anti-tumor activity toward a variety of tumor cells without inducing cytotoxicity in normal cells (You et al., 2013). In the cactus (*Opuntia dilleniid*), polysaccharides were found that can induce S-phase arrest and block the growth of SK-MES-1 cells, possibly through increased levels of P53 and phosphatase and tensin homolog deleted on chromosome 10 protein (PTEN) (Li et al., 2014). The screening and discovery of new anti-tumor polysaccharides represents an ongoing pursuit for many research groups, with the most common direct tumor-killing mechanisms of anti-tumor polysaccharides involving cell cycle arrest, blocking angiogenesis, and inducing apoptosis. In addition, some polysaccharides act through immunomodulation to indirectly induce tumor killing (Khan T. et al., 2019; Lu et al., 2020).

Several studies have described antiviral effects of both natural and chemically modified polysaccharides. For example, Witvrouw et al. (1994) observed antiviral activity by a sulfated polysaccharide isolated from the red seaweed (*Aghardhiella tenera*) *in vitro* and found that it could inhibit the cytopathic effects of human immunodeficiency virus type 1 (HIV-1) and type 2 (HIV-2), as well as against other enveloped viruses. Examination of the inhibitory effects of *Glycyrrhiza* polysaccharide (GPS) on bovine immunodeficiency virus (BIV), adenovirus type III (AdVIII), and coxsackie virus (CBV3) revealed that GPS could inhibit BIV to some extent, but showed obvious inhibition or direct inactivation of AdVIII and CBV3 (Wang Y. et al., 2020). Further technological advances in plant polysaccharide research will enable more comprehensive screening for effective anti-tumor and antiviral polysaccharide drugs.

## 3. Prebiotic effects of various sources of polysaccharides on gut microbiota

The adult human intestinal tract harbors an estimated stable community of 39 trillion microbial cells, which has been recognized as a diverse and dynamic ecosystem containing bacteria, fungi, protozoa, and viruses (Rajakovich and Balskus, 2019). Gut bacterial communities are generally comprised of six major phyla, including Firmicutes, Bacteroidetes, Proteobacteria, Actinobacteria, Verrucomicrobia, and Fusobacteria (Gong et al., 2020), which collectively contribute to protecting the intestinal barrier, preventing pathogen invasion, participating in vitamin synthesis (Vitamin K, Vitamin B), host metabolism, and other functions related to nutrient uptake (Cresci and Bawden, 2015). In addition, mutual interactions between gut microbiota and their host were found to provide important functions in host health, and dysbiosis in gut microbiota has been linked to inflammatory bowel disease, obesity, allergies, and psychological disorders (Coyte et al., 2015). Patterson et al. (2016) proposed a possible association between gut microbiota and obesity and metabolic syndrome, which are both accompanied by an increased ratio of Firmicutes to Bacteroidetes in obese phenotypes patients, compared with that in non-obese individuals (Patterson et al., 2016). Other studies have indicated that gut microbiota can influence the development of neurological disorders as autism, depression, Alzheimer’s disease, and Parkinson’s disease (Dinan and Cryan, 2017).

**Figure 4:**
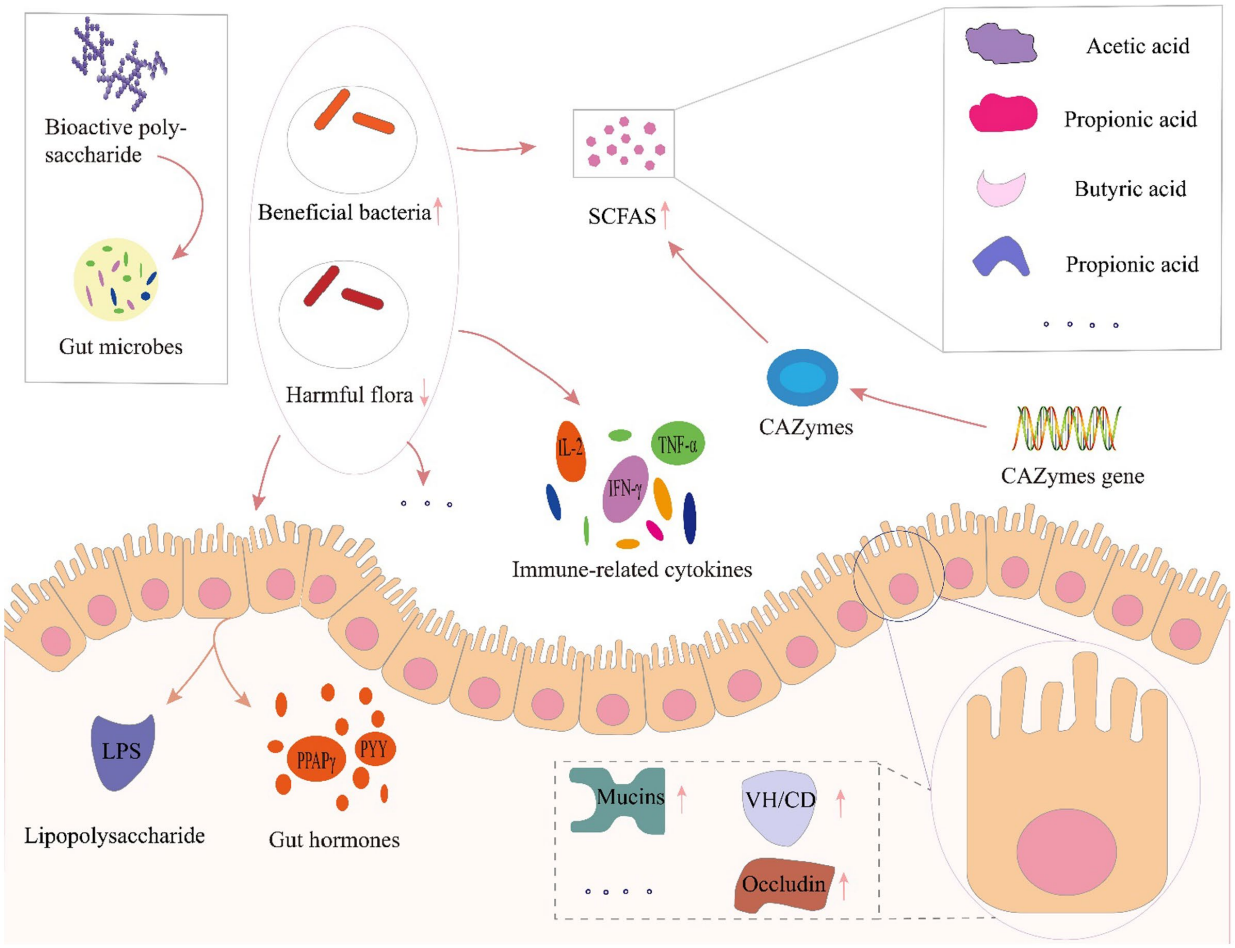
Prebiotic effects of polysaccharides toward gut microbial modulation. LPS, lipopolysaccharide; VH/CD, villus height/crypt depth; CAZymes, carbohydrate-active enzymes; SCFA, short-chain fatty acids; PPARγ, peroxisome proliferator-activated receptor-γ; PYY, peptide tyrosine; IL-2, interleukin-2; IFN-γ, interferon-γ; TNF-α, tumor necrosis factor-α.

Several factors, including infant delivery modes and feeding patterns, host, diet, antibiotic usage, and other factors have all been reported to affect gut microbiota composition and diversity (Cresci and Bawden, 2015). Among these factors, host and diet exert a particularly strong influence on gut microbiota. Our own previous study identified clear differences in gut microbiota driven by dietary variation among people living in close regional proximity (Liu et al., 2020). Furthermore, dietary intervention was recently recognized as a reliable strategy for altering gut microbiota and enhancing gut function, especially through prebiotics. Prebiotics are food components that may promote health in the host by exert activity in the gastrointestinal microbiota, such as fructose oligosaccharides, arabino-oligosaccharides, isomalt oligosaccharides, polyphenols and polyunsaturated fatty acids and etc. (Gibson et al., 2004, 2017). Among these prebiotics, dietary polysaccharides are relatively large macromolecules that are difficult to digest and absorbed, but can serve as carbon sources that potentially enrich beneficial gut microbes, or regulate microbial growth, activity, and metabolite production (Koropatkin et al., 2012; Chang et al., 2015; Wang et al., 2018). Furthermore, the polysaccharides affected gut microbiota modulation *via* enrichment for beneficial taxa and suppression of potential pathogens, leading to increased microbial expression of carbohydrate-active enzymes and enhanced short chain fatty acid production. In addition, polysaccharide mediated improvements in gut function such as influencing immune-related cytokines and hormone secretion in host intestinal epithelial cells and maintaining gut barrier function (Figure 4).

Plant polysaccharides have been frequently proposed to serve as regulators of gut microecology (Fang et al., 2019), and have been shown to regulate the diversity and structure of gut microbiota, thus altering metabolic functions in the gastrointestinal tract (Zhao et al., 2021). Oral administration of polysaccharides from *Ziziphus Jujuba* cv. Pozao in cyclophosphamide-induced mice led to enrichment of Firmicutes and decreased Bacteroidetes, with genus-level increases in the relative abundance of *Bacteroidales-S24-7-group*, *Lachnospiraceae*, *Alloprevotella*, *Alistipes,* and *Bacteroides* (Han et al., 2020). Chen and colleagues showed that *Sarcodon aspratus* polysaccharides could serve as a prebiotic treatment to prevent obesity in mice fed with a high-fat diet, and led to increased relative abundance of *Lactobacillus*, *Bacteroides* and *Akkermansia*, decreased Firmicutes-to-Bacteroidetes ratio, inhibitory effects on immune cells activation and adipocyte differentiation in adipose tissues (Chen et al., 2020). *Poria cocos* polysaccharides were shown to alleviate intestinal mucosal injury, improve intestinal integrity, and restore the composition and structure of gut microbiota following dysbiosis by promoting the proliferation of beneficial bacteria in Ob/Ob Mice (Sun et al., 2019). Li et al. (2020) investigated the hypoglycemic and hypolipidemic effects of tea polysaccharides, potentially mediated by changes in gut microbiota and metabolism, in a rat model of type 2 diabetes. They stated that treatment with tea polysaccharides restored some specific bacterial taxa, such as *Lachnospira*, *Victivallis*, *Roseburia*, and *Fluviicola* in diabetic rats, whereas *Bacteroides* was decreased (Li et al., 2020). Taken together, polysaccharides play an important role in improving body health by increasing the enrichment of beneficial bacteria and reducing the proximity of harmful bacteria.

In addition, gut microbiota could produce short-chain fatty acids (SCFAs) and other metabolites, such as acetic acid, propionic acid, butyric acid, lactic acid and succinic acids, etc. These SCFAs can, in turn, affect gut functions to jointly regulate health (Wu Y et al., 2019). SCFAs are the final product of polysaccharide fermentation by gut microbiota (Mitsou et al., 2020), and are well-known to contribute to maintaining epithelial cell growth in the colon, modulate host metabolism, participate in immune regulation of the intestinal system, and play an indispensable role in maintaining the homeostasis of human gut microbiota (Morrison and Preston, 2016). In a study by Guo et al., treatment with polysaccharide from hawthorn (*Crataegus monogyna*) by gavage ameliorated inflammation symptoms in mice with dextran sulfate sodium (DSS)-induced colitis through significantly increased production of total SCFAs and acetic acid, attributable to greater relative abundance of *Alestipes* and *Odoribacter* (Guo et al., 2021b). Investigation of *Cyclocarya paliurus* polysaccharides in type 2 diabetic rat models. Suggested that these molecules could enrich for SCFA-producing bacteria, leading to elevated SCFA production and upregulation of associated sensory mediators that alleviate symptoms of type 2 diabetes (Yao et al., 2020). *β*-glucans, inulin, and oligofructose have all been found to significantly increase butyric acid production, which serve as a nutrient source for intestinal epithelial cells, reduces pH in the lumen, and provides energy for human activities (Song et al., 2014; Kim and Jazwinski, 2018), whereas polysaccharides such as α-glucan, fructan, and arabinoxylan can reportedly increase intestinal acetic acid levels (Shang et al., 2018). Insoluble polysaccharide isolated from the sclerotium of *Poria cocos*, as a prebiotic, increased the abundance of butyrate-producing bacteria such as *Lachnospiracea* and *Clostridium*, leading to an increase in the level of butyrate and improvement of gut mucosal integrity and activated the intestinal PPAR-γ pathway, significantly improving glucose and lipid metabolism and alleviating hepatic steatosis in ob/ob mice, suggesting its potential for the treatment of metabolic diseases (Sun et al., 2019). As one of the important natural polysaccharides source, marine polysaccharides were also well studied recently. Lentinan and sea anemone polysaccharides were recently reported to facilitate the production of total SCFAs, maintain gut homeostasis, and provide energy (Wang et al., 2018). In addition, polysaccharides from tea, *C. sinensis,* can also reduce blood glucose and lipid levels, promote SCFA production, attenuate insulin resistance, confer protective effects against pancreatic damage in type 2 diabetic rat model (Li et al., 2020).

In addition to regulating SCFA production, study showed that plant polysaccharides can up-regulate the expression of genes encoding carbohydrate-active enzymes (CAZymes), enhancing CAZyme activity and thus leads to higher SCFA production and increased tight junction protein expression with concurrent suppression of metabolic endotoxemia and decreased expression of inflammatory factors (Nguyen et al., 2016). Guo et al. (2021a) reported that *Ganoderma lucidum* polysaccharide could also upregulate CAZyme expression, especially glycoside hydrolases, polysaccharide lyases, glycosyltransferases, and carbohydrate esterases, leading to improved health.

Furthermore, polysaccharides can facilitate repair of damaged intestinal barrier to ensure the integrity of intestinal structures (Liang J. et al., 2021). Zhou et al. (2021) conducted co-treatment of American ginseng polysaccharide and ginsenoside altered uric acid, xanthurenic acid, acylcarnitine and restored the morphology of intestine. Specifically, the co-treatment resulted in an up-regulation of the villus height (VH)/crypt depth (CD) ratio, as well as an increase in the areas of mucins expression, quantity of goblet cells and expression of tight junction proteins (ZO-1, occludin) and then protecting the intestinal barrier (Zhou et al., 2021). Additionally, study of Yu et al. (2021) found that *Cyclocarya paliurus* polysaccharides dramatically increased the intestine antioxidant defense of CTX-induced mice, repaired the intestinal barrier by restoring the length of villi and depth of crypt, up-regulating the expression of tight junction proteins, shifting the composition and diversity of the gut microbiota, and regulating the relative abundances of specific taxa, then restoring intestinal mucosal barrier function (Yu et al., 2021).

Additionally, polysaccharide mediated improvements in gut function such as influencing immune-related cytokines and hormone secretion. Study of *Lycium barbarum* polysaccharide also suggested that polysaccharide treatment could protect immune organs, enhance the production of immune-related cytokines (IL-2, IL-6, IL-1β, TNF-α, and IFN-γ) and prevent the hepatotoxicity in cyclophosphamide (CTX)-induced mice (Ding et al., 2019). In immunosuppressed mice, *Cordyceps sinensis* polysaccharide was found to modulate gut microbiota, alleviate gut injury, and regulate the balance of T helper (Th)1/Th2 cells (Chen S. et al., 2021). Khan I. et al. (2019) reported that the treatment of *Ganoderma lucidum* polysaccharides markedly promoted SCFA produced bacteria and abridged sulfate-reducing bacteria in a time-dependent manner, altered expressions of histone deacetylases, anti-cancer gut hormone PYY, and PPAPγ in ApcMin/+ mice. Collectively, both *in vitro* and animal experiments applied for polysaccharide from different sources has been proved to have gut modulation capacity as prebiotic, however, clinical application of well recognized polysaccharides could help further understanding the actual response of human gut microbiota. These studies collectively indicate that plant polysaccharides share a complex relationship with gut microbes, and further study is required to reveal the full scope of mechanisms. In addition, gut regulation by postbiotic as secreted polysaccharides and extracellular polysaccharides has aroused attention recently. In several studies, the lactic acid bacteria-exopolysaccharides showed anti-oxidative and immunomodulatory activities as well as gut microbiota regulation effect (Xu R. et al., 2019; Xu Y. et al., 2019; Nataraj et al., 2020; Wang et al., 2020a). Further research into the biological activities of these metabolites is expected to reveal novel uses for postbiotics in medicine and beyond.

In general, the influence of polysaccharides on gut microbes can be summarized in three main aspects. Firstly, polysaccharides can attenuate damage to intestinal mucosa, alleviate intestinal inflammation, facilitate repair of damaged intestinal barrier, and ensure the integrity of the intestinal structure. Second, polysaccharides can alter intestinal microbial community composition and function, enriching beneficial bacteria while suppressing the proliferation of potential pathogens, consequently improving the relative content of various intestinal metabolites, especially SCFAs, that positively impact overall health. Third, polysaccharides can directly regulate gut function, such as modulating the secretion of interleukins and hormones in intestinal epithelial cells, or activating microbial CAZyme expression, which can reduce the likelihood of gut disease. However, most of these interventional studies are based on *in vitro* experiments or model animals, and thus clinical trials examining the effects of polysaccharide-based treatments in gut dysbiosis could improve our understanding of the interactions between polysaccharides, gut microbiota, and gastrointestinal function in humans.

## 4. Conclusion

Dietary polysaccharides have been shown to exert a range of biological activities, including anticoagulation, anti-inflammatory, immunomodulatory, hypoglycemic, antitumor, and antioxidant effects that impact gut health, in addition to modulating gut function and microbiota composition. These diverse functions are largely dependent on the primary structure and functional groups of the saccharide monomers. Ongoing innovations in data mining and compound screening continually expand the range of available bioactive polysaccharides, and drive the development of their pharmacological applications. It is noteworthy that these bioactive polysaccharides are not strictly limited to plants, and have been screened from fungi and algal sources, suggesting that there are likely a multitude of such metabolites with potential health benefits that are yet to be discovered in nature.

## Author contributions

BL and ZL: conceptualization. YN and WL: writing – original draft preparation. BL, ZL, XF, DWX, DW, and HW: revising entire manuscript draft. BL, ZL, and XF: supervising, reviewing and editing final version of article. All authors have read and agreed to the published version of the manuscript.

## Funding

The study was supported by Special Item of Regional Collaborative Innovation in Tibet Autonomous Region (QYXTZX-LS2021-01) and Key Research and Development Pro-gram in Tibet Autonomous Region (XZ202201ZY0004N and XZ202301ZY0018N).

## Conflict of interest

The authors declare that the research was conducted in the absence of any commercial or financial relationships that could be construed as a potential conflict of interest.

## Publisher’s note

All claims expressed in this article are solely those of the authors and do not necessarily represent those of their affiliated organizations, or those of the publisher, the editors and the reviewers. Any product that may be evaluated in this article, or claim that may be made by its manufacturer, is not guaranteed or endorsed by the publisher.

## References

[ref1] Björkstén B. Sepp E. Julge K. Voor T. Mikelsaar M. (2001). Allergy development and the intestinal microflora during the first year of life. J. Allergy Clin. Immunol. 108, 516–520. doi: 10.1067/mai.2001.118130, PMID: 11590374

[ref2] Bunyatyan N. D. Bukhtiyarova I. P. Drogovoz S. M. Kononenko A. V. Olefir Y. V. Prokof’ev A. B.. (2017). Influence of human biorhythms on the blood glucose level and the efficacy of hypoglycemic drugs (review). Pharm. Chem. J. 51, 399–401. doi: 10.1007/s11094-017-1621-4, PMID: 9550123

[ref3] Cao Y.-Y. Ji Y.-H. Liao A.-M. Huang J.-H. Thakur K. Li X.-L.. (2020). Effects of sulfated, phosphorylated and carboxymethylated modifications on the antioxidant activities *in-vitro* of polysaccharides sequentially extracted from *Amana edulis*. Int. J. Biol. Macromol. 146, 887–896. doi: 10.1016/j.ijbiomac.2019.09.211, PMID: 31669658

[ref4] Chaisuwan W. Phimolsiripol Y. Chaiyaso T. Techapun C. Leksawasdi N. Jantanasakulwong K.. (2021). The antiviral activity of bacterial, fungal, and algal polysaccharides as bioactive ingredients: potential uses for enhancing immune systems and preventing viruses. Front. Nutr. 8:772033. doi: 10.3389/fnut.2021.772033, PMID: 34805253 PMC8602887

[ref5] Chang C.-J. Lin C.-S. Lu C.-C. Martel J. Ko Y.-F. Ojcius D. M.. (2015). *Ganoderma lucidum* reduces obesity in mice by modulating the composition of the gut microbiota. Nat. Commun. 6:7489. doi: 10.1038/ncomms8489, PMID: 26102296 PMC4557287

[ref6] Chen G. Chen R. Chen D. Ye H. Hu B. Zeng X.. (2019). Tea polysaccharides as potential therapeutic options for metabolic diseases. J. Agric. Food Chem. 67, 5350–5360. doi: 10.1021/acs.jafc.8b05338, PMID: 30474370

[ref7] Chen F. Huang G. Yang Z. Hou Y. (2019). Antioxidant activity of *Momordica charantia* polysaccharide and its derivatives. Int. J. Biol. Macromol. 138, 673–680. doi: 10.1016/j.ijbiomac.2019.07.129, PMID: 31344411

[ref8] Chen J. Liu J. Yan C. Zhang C. Pan W. Zhang W.. (2020). *Sarcodon aspratus* polysaccharides ameliorated obesity-induced metabolic disorders and modulated gut microbiota dysbiosis in mice fed a high-fat diet. Food Funct. 11, 2588–2602. doi: 10.1039/C9FO00963A, PMID: 32154540

[ref9] Chen X. Qian L. Wang B. Zhang Z. Liu H. Zhang Y.. (2019). Synergistic Hypoglycemic effects of pumpkin polysaccharides and Puerarin on type II diabetes mellitus mice. Molecules 24:955. doi: 10.3390/molecules24050955, PMID: 30857163 PMC6429091

[ref10] Chen S. Wang J. Fang Q. Dong N. Fang Q. Cui S. W.. (2021). A polysaccharide from natural *cordyceps sinensis* regulates the intestinal immunity and gut microbiota in mice with cyclophosphamide-induced intestinal injury. Food Funct. 12, 6271–6282. doi: 10.1039/d1fo00596k, PMID: 34105571

[ref11] Chen Y.-C. Wu Y.-J. Hu C.-Y. (2019). Monosaccharide composition influence and immunomodulatory effects of probiotic exopolysaccharides. Int. J. Biol. Macromol. 133, 575–582. doi: 10.1016/j.ijbiomac.2019.04.109, PMID: 31004639

[ref12] Chen C. Xie X. Li X. (2021). Immunomodulatory effects of four polysaccharides purified from *Erythronium sibiricum* bulb on macrophages. Glycoconj. J. 38, 517–525. doi: 10.1007/s10719-021-10005-z, PMID: 34117963

[ref13] Chiu Y.-H. Lin S.-L. Tsai J.-J. Lin M.-Y. (2014). Probiotic actions on diseases: implications for therapeutic treatments. Food Funct. 5, 625–634. doi: 10.1039/c3fo60600g, PMID: 24549263

[ref14] Coyte K. Z. Schluter J. Foster K. R. (2015). The ecology of the microbiome: networks, competition, and stability. Science 350, 663–666. doi: 10.1126/science.aad2602, PMID: 26542567

[ref15] Cresci G. A. Bawden E. (2015). Gut microbiome: what we do and don’t know. Nutr. Clin. Pract. 30, 734–746. doi: 10.1177/0884533615609899, PMID: 26449893 PMC4838018

[ref16] Cui M. Zhou R. Wang Y. Zhang M. Liu K. Ma C. (2020). Beneficial effects of sulfated polysaccharides from the red seaweed *Gelidium pacificum* Okamura on mice with antibiotic-associated diarrhea. Food Funct. 11, 4625–4637. doi: 10.1039/D0FO00598C, PMID: 32400829

[ref17] D’Ayala G. Malinconico M. Laurienzo P. (2008). Marine derived polysaccharides for biomedical applications: chemical modification approaches. Molecules 13, 2069–2106. doi: 10.3390/molecules13092069, PMID: 18830142 PMC6245343

[ref18] de Vrese M. Schrezenmeir J. (2008). Probiotics, prebiotics, and synbiotics. Adv. Biochem. Eng. Biotechnol. 111, 1–66. doi: 10.1007/10_2008_097, PMID: 18461293

[ref19] Dinan T. G. Cryan J. F. (2017). Gut instincts: microbiota as a key regulator of brain development, ageing and neurodegeneration. J. Physiol. 595, 489–503. doi: 10.1113/JP273106, PMID: 27641441 PMC5233671

[ref20] Ding Y. Yan Y. Chen D. Ran L. Mi J. Lu L.. (2019). Modulating effects of polysaccharides from the fruits of *Lycium barbarum* on the immune response and gut microbiota in cyclophosphamide-treated mice. Food Funct. 10, 3671–3683. doi: 10.1039/C9FO00638A, PMID: 31168539

[ref21] Fang Q. Hu J. Nie Q. Nie S. (2019). Effects of polysaccharides on glycometabolism based on gut microbiota alteration. Trends Food Sci. Technol. 92, 65–70. doi: 10.1016/j.tifs.2019.08.015, PMID: 33072135

[ref22] Fang Y. Z. Yang S. Wu G. (2002). Free radicals, antioxidants, and nutrition. Nutrition 18, 872–879. doi: 10.1016/S0899-9007(02)00916-4, PMID: 12361782

[ref23] Fridovich I. (1999). Fundamental aspects of reactive oxygen species, or what’s the matter with oxygen? Ann. N. Y. Acad. Sci. 893, 13–18. doi: 10.1111/j.1749-6632.1999.tb07814.x, PMID: 10672226

[ref24] Fu Y.-P. Li C.-Y. Peng X. Zou Y.-F. Rise F. Paulsen B. S.. (2022). Polysaccharides from *Aconitum carmichaelii* leaves: structure, immunomodulatory and anti-inflammatory activities. Carbohydr. Polym. 291:119655. doi: 10.1016/j.carbpol.2022.119655, PMID: 35698356

[ref25] Fuke N. Nagata N. Suganuma H. Ota T. (2019). Regulation of gut microbiota and metabolic endotoxemia with dietary factors. Nutrients 11:2277. doi: 10.3390/nu11102277, PMID: 31547555 PMC6835897

[ref26] Gabriela das Chagas Faustino Alves M. Almeida-Lima J. Paiva A. A. O. Leite E. L. Rocha H. A. O. (2016). Extraction process optimization of sulfated galactan-rich fractions from *Hypnea musciformis* in order to obtain antioxidant, anticoagulant, or immunomodulatory polysaccharides. J. Appl. Phycol. 28, 1931–1942. doi: 10.1007/s10811-015-0705-3, PMID: 37151649

[ref27] Ganesan K. Xu B. (2019). Anti-diabetic effects and mechanisms of dietary polysaccharides. Molecules 24:E2556. doi: 10.3390/molecules24142556, PMID: 31337059 PMC6680889

[ref28] Gao Z. Zhang C. Tian W. Liu K. Hou R. Yue C.. (2017). The antioxidative and hepatoprotective effects comparison of Chinese angelica polysaccharide (CAP) and selenizing CAP (sCAP) in CCl4 induced hepatic injury mice. Int. J. Biol. Macromol. 97, 46–54. doi: 10.1016/j.ijbiomac.2017.01.013, PMID: 28064055

[ref29] Gibson G. R. Hutkins R. Sanders M. E. Prescott S. L. Reimer R. A. Salminen S. J.. (2017). Expert consensus document: the International Scientific Association for Probiotics and Prebiotics (ISAPP) consensus statement on the definition and scope of prebiotics. Nat. Rev. Gastroenterol. Hepatol. 14, 491–502. doi: 10.1038/nrgastro.2017.75, PMID: 28611480

[ref30] Gibson G. R. Probert H. M. Loo J. V. Rastall R. A. Roberfroid M. B. (2004). Dietary modulation of the human colonic microbiota: updating the concept of prebiotics. Nutr. Res. Rev. 17, 259–275. doi: 10.1079/NRR200479, PMID: 19079930

[ref31] Gong W.-P. Han R. Li H.-S. Song J.-M. Yan H.-F. Li G.-Y.. (2017). Agronomic traits and molecular marker identification of wheat- *Aegilops caudata* addition lines. Front. Plant Sci. 8:1743. doi: 10.3389/fpls.2017.01743, PMID: 29075275 PMC5644244

[ref32] Gong X. Li X. Bo A. Shi R.-Y. Li Q.-Y. Lei L.-J.. (2020). The interactions between gut microbiota and bioactive ingredients of traditional Chinese medicines: a review. Pharmacol. Res. 157:104824. doi: 10.1016/j.phrs.2020.104824, PMID: 32344049

[ref33] Govindan S. Shanmugam J. Rajendran G. Ramani P. Unni D. Venkatachalam B.. (2023). Antidiabetic activity of polysaccharide from *Hypsizygus ulmarius* in streptozotocin-nicotinamide induced diabetic rats. Bioact. Carbohydr. Diet. Fibre 29:100350. doi: 10.1016/j.bcdf.2023.100350

[ref34] Guo C. Guo D. Fang L. Sang T. Wu J. Guo C.. (2021a). *Ganoderma lucidum* polysaccharide modulates gut microbiota and immune cell function to inhibit inflammation and tumorigenesis in colon. Carbohydr. Polym. 267:118231. doi: 10.1016/j.carbpol.2021.118231, PMID: 34119183

[ref35] Guo L. Ma R. Sun H. Raza A. Tang J. Li Z. (2018). Anti-inflammatory activities and related mechanism of polysaccharides isolated from *Sargentodoxa cuneata*. Chem. Biodivers. 15:e1800343. doi: 10.1002/cbdv.201800343, PMID: 30153400

[ref36] Guo C. Wang Y. Zhang S. Zhang X. Du Z. Li M.. (2021b). *Crataegus pinnatifida* polysaccharide alleviates colitis via modulation of gut microbiota and SCFAs metabolism. Int. J. Biol. Macromol. 181, 357–368. doi: 10.1016/j.ijbiomac.2021.03.137, PMID: 33774071

[ref37] Han X. Bai B. Zhou Q. Niu J. Yuan J. Zhang H.. (2020). Dietary supplementation with polysaccharides from *Ziziphus Jujuba* cv. Pozao intervenes in immune response via regulating peripheral immunity and intestinal barrier function in cyclophosphamide-induced mice. Food Funct. 11, 5992–6006. doi: 10.1039/D0FO00008F, PMID: 32697211

[ref38] Han Y. Nan S. Fan J. Chen Q. Zhang Y. (2019). *Inonotus obliquus* polysaccharides protect against Alzheimer’s disease by regulating Nrf2 signaling and exerting antioxidative and antiapoptotic effects. Int. J. Biol. Macromol. 131, 769–778. doi: 10.1016/j.ijbiomac.2019.03.033, PMID: 30878614

[ref39] Hasan A. Waibhaw G. Tiwari S. Dharmalingam K. Shukla I. Pandey L. M. (2017). Fabrication and characterization of chitosan, polyvinylpyrrolidone, and cellulose nanowhiskers nanocomposite films for wound healing drug delivery application. J. Biomed. Mater. Res. A 105, 2391–2404. doi: 10.1002/jbm.a.36097, PMID: 28445626

[ref40] Hatcher E. Sawen E. Widmalm G. MacKerell A. D. J. (2011). Conformational properties of methyl β-maltoside and methyl α- and β-cellobioside disaccharides. J. Phys. Chem. B 115, 597–608. doi: 10.1021/jp109475p, PMID: 21158455 PMC3077104

[ref41] Jayachandran M. Wu Z. Ganesan K. Khalid S. Chung S. M. Xu B. (2019). Isoquercetin upregulates antioxidant genes, suppresses inflammatory cytokines and regulates AMPK pathway in streptozotocin-induced diabetic rats. Chem. Biol. Interact. 303, 62–69. doi: 10.1016/j.cbi.2019.02.017, PMID: 30817903

[ref42] Kalinina T. S. Zlenko D. V. Kiselev A. V. Litvin A. A. Stovbun S. V. (2020). Antiviral activity of the high-molecular-weight plant polysaccharides (Panavir®). Int. J. Biol. Macromol. 161, 936–938. doi: 10.1016/j.ijbiomac.2020.06.031, PMID: 32534094 PMC7287457

[ref43] Kemp M. M. Kumar A. Mousa S. Dyskin E. Yalcin M. Ajayan P.. (2009). Gold and silver nanoparticles conjugated with heparin derivative possess anti-angiogenesis properties. Nanotechnology 20:455104. doi: 10.1088/0957-4484/20/45/455104, PMID: 19822927

[ref44] Khan T. Date A. Chawda H. Patel K. (2019). Polysaccharides as potential anticancer agents-a review of their progress. Carbohydr. Polym. 210, 412–428. doi: 10.1016/j.carbpol.2019.01.064, PMID: 30732778

[ref45] Khan I. Huang G. Li X.-A. Liao W. Leong W. K. Xia W.. (2019). Mushroom polysaccharides and jiaogulan saponins exert cancer preventive effects by shaping the gut microbiota and microenvironment in ApcMin/+ mice. Pharmacol. Res. 148:104448. doi: 10.1016/j.phrs.2019.104448, PMID: 31499195

[ref46] Kiddane A. T. Kim G.-D. (2021). Anticancer and immunomodulatory effects of polysaccharides. Nutr. Cancer 73, 2219–2231. doi: 10.1080/01635581.2020.1861310, PMID: 33356601

[ref47] Kim S. Jazwinski S. M. (2018). The gut microbiota and healthy aging: a mini-review. Gerontology 64, 513–520. doi: 10.1159/000490615, PMID: 30025401 PMC6191326

[ref48] Koropatkin N. M. Cameron E. A. Martens E. C. (2012). How glycan metabolism shapes the human gut microbiota. Nat. Rev. Microbiol. 10, 323–335. doi: 10.1038/nrmicro2746, PMID: 22491358 PMC4005082

[ref49] Kouakou K. Schepetkin I. A. Yapi A. Kirpotina L. N. Jutila M. A. Quinn M. T. (2013). Immunomodulatory activity of polysaccharides isolated from *Alchornea cordifolia*. J. Ethnopharmacol. 146, 232–242. doi: 10.1016/j.jep.2012.12.037, PMID: 23291534 PMC3577965

[ref50] Lafond M. Sulzenbacher G. Freyd T. Henrissat B. Berrin J.-G. Garron M.-L. (2016). The quaternary structure of a glycoside hydrolase dictates specificity toward β-glucans. J. Biol. Chem. 291, 7183–7194. doi: 10.1074/jbc.M115.695999, PMID: 26755730 PMC4807298

[ref51] Li W. Deng Y. Chu Q. Zhang P. (2019). Gut microbiome and cancer immunotherapy. Cancer Lett. 447, 41–47. doi: 10.1016/j.canlet.2019.01.015, PMID: 30684593

[ref52] Li H. Fang Q. Nie Q. Hu J. Yang C. Huang T.. (2020). Hypoglycemic and hypolipidemic mechanism of tea polysaccharides on type 2 diabetic rats via gut microbiota and metabolism alteration. J. Agric. Food Chem. 68, 10015–10028. doi: 10.1021/acs.jafc.0c01968, PMID: 32811143

[ref53] Li J.-P. Kusche-Gullberg M. (2016). Heparan Sulfate: biosynthesis, structure, and function. Int. Rev. Cell Mol. Biol. 325, 215–273. doi: 10.1016/bs.ircmb.2016.02.009 27241222

[ref54] Li W. Wu D. Wei B. Wang S. Sun H. Li X.. (2014). Anti-tumor effect of cactus polysaccharides on lung squamous carcinoma cells (SK-MES-1). Afr. J. Tradit. Complement. Altern. Med. 11, 99–104. doi: 10.4314/ajtcam.v11i5.16, PMID: 25395712 PMC4202525

[ref55] Li S. Xiong Q. Lai X. Li X. Wan M. Zhang J.. (2016). Molecular modification of polysaccharides and resulting bioactivities. Compr. Rev. Food Sci. Food Saf. 15, 237–250. doi: 10.1111/1541-4337.12161, PMID: 33371599

[ref56] Liang Q. Dong J. Wang S. Shao W. Ahmed A. F. Zhang Y.. (2021). Immunomodulatory effects of *Nigella sativa* seed polysaccharides by gut microbial and proteomic technologies. Int. J. Biol. Macromol. 184, 483–496. doi: 10.1016/j.ijbiomac.2021.06.118, PMID: 34166694

[ref57] Liang J. Zhang M. Wang X. Ren Y. Yue T. Wang Z.. (2021). Edible fungal polysaccharides, the gut microbiota, and host health. Carbohydr. Polym. 273:118558. doi: 10.1016/j.carbpol.2021.118558, PMID: 34560969

[ref58] Liao W. Luo Z. Liu D. Ning Z. Yang J. Ren J. (2015). Structure characterization of a novel polysaccharide from *Dictyophora indusiata* and its macrophage immunomodulatory activities. J. Agric. Food Chem. 63, 535–544. doi: 10.1021/jf504677r, PMID: 25525995

[ref59] Lin G. Luo D. Liu J. Wu X. Chen J. Huang Q.. (2018). Hepatoprotective effect of polysaccharides isolated from *Dendrobium officinale* against acetaminophen-induced liver injury in mice via regulation of the Nrf2-Keap1 Signaling pathway. Oxidative Med. Cell. Longev. 2018:6962439. doi: 10.1155/2018/6962439, PMID: 30116489 PMC6079321

[ref60] Lin Y. Pi J. Jin P. Liu Y. Mai X. Li P.. (2022). Enzyme and microwave co-assisted extraction, structural characterization and antioxidant activity of polysaccharides from *purple-heart radish*. Food Chem. 372:131274. doi: 10.1016/j.foodchem.2021.131274, PMID: 34638061

[ref61] Liu Y. Chen D. You Y. Zeng S. Hu Y. Duan X.. (2016a). Structural characterization and antidiabetic activity of a glucopyranose-rich heteropolysaccharide from *Catathelasma ventricosum*. Carbohydr. Polym. 149, 399–407. doi: 10.1016/j.carbpol.2016.04.106, PMID: 27261764

[ref62] Liu Z. Jiao Y. Wang Y. Zhou C. Zhang Z. (2008). Polysaccharides-based nanoparticles as drug delivery systems. Adv. Drug Deliv. Rev. 60, 1650–1662. doi: 10.1016/j.addr.2008.09.001, PMID: 18848591

[ref63] Liu Y. Li X. Xie C. Luo X. Bao Y. Wu B.. (2016b). Prevention effects and possible molecular mechanism of mulberry leaf extract and its formulation on rats with insulin-insensitivity. PLoS One 11:e0152728. doi: 10.1371/journal.pone.0152728, PMID: 27054886 PMC4824359

[ref64] Liu K. Zhang Y. Li Q. Li H. Long D. Yan S.. (2020). Ethnic differences shape the alpha but not Beta diversity of gut microbiota from school children in the absence of environmental differences. Microorganisms 8:254. doi: 10.3390/microorganisms8020254, PMID: 32075068 PMC7074779

[ref65] Lu J. He R. Sun P. Zhang F. Linhardt R. J. Zhang A. (2020). Molecular mechanisms of bioactive polysaccharides from *Ganoderma lucidum* (Lingzhi), a review. Int. J. Biol. Macromol. 150, 765–774. doi: 10.1016/j.ijbiomac.2020.02.035, PMID: 32035956

[ref66] Luo Y. Peng B. Wei W. Tian X. Wu Z. (2019). Antioxidant and anti-diabetic activities of polysaccharides from guava leaves. Molecules 24:1343. doi: 10.3390/molecules24071343, PMID: 30959759 PMC6479919

[ref67] Ma L. Xu G. B. Tang X. Zhang C. Zhao W. Wang J.. (2020). Anti-cancer potential of polysaccharide extracted from hawthorn (*Crataegus*.) on human colon cancer cell line HCT116 via cell cycle arrest and apoptosis. J. Funct. Foods 64:103677. doi: 10.1016/j.jff.2019.103677

[ref68] Maji B. (2019). Introduction to natural polysaccharides. Funct. Polysacch. Biomed. Applic. 1–31. doi: 10.1016/B978-0-08-102555-0.00001-7

[ref69] Mata Y. N. Torres E. Blázquez M. L. Ballester A. González F. Muñoz J. A. (2009). Gold(III) biosorption and bioreduction with the brown alga *Fucus vesiculosus*. J. Hazard. Mater. 166, 612–618. doi: 10.1016/j.jhazmat.2008.11.064, PMID: 19124199

[ref70] Matsubara K. Matsuura Y. Bacic A. Liao M. Hori K. Miyazawa K. (2001). Anticoagulant properties of a sulfated galactan preparation from a marine green alga, *Codium cylindricum*. Int. J. Biol. Macromol. 28, 395–399. doi: 10.1016/S0141-8130(01)00137-4, PMID: 11325427

[ref71] Min Z. Xiaona H. Aziz T. Jian Z. Zhennai Y. (2020). Exopolysaccharides from *Lactobacillus plantarum* YW11 improve immune response and ameliorate inflammatory bowel disease symptoms. Acta Biochim. Pol. 67, 485–493. doi: 10.18388/abp.2020_5371, PMID: 33332076

[ref72] Mirzadeh M. Keshavarz Lelekami A. Khedmat L. (2021). Plant/algal polysaccharides extracted by microwave: a review on hypoglycemic, hypolipidemic, prebiotic, and immune-stimulatory effect. Carbohydr. Polym. 266:118134. doi: 10.1016/j.carbpol.2021.118134, PMID: 34044950

[ref73] Mitsou E. K. Saxami G. Stamoulou E. Kerezoudi E. Terzi E. Koutrotsios G.. (2020). Effects of rich in Β-glucans edible mushrooms on aging gut microbiota characteristics: an *in vitro* study. Molecules 25:E2806. doi: 10.3390/molecules25122806, PMID: 32570735 PMC7355846

[ref74] Morrison D. J. Preston T. (2016). Formation of short chain fatty acids by the gut microbiota and their impact on human metabolism. Gut Microbes 7, 189–200. doi: 10.1080/19490976.2015.1134082, PMID: 26963409 PMC4939913

[ref75] Mousavi S. Moradi M. Khorshidahmad T. Motamedi M. (2015). Anti-inflammatory effects of heparin and its derivatives: a systematic review. Adv. Pharmacol. Sci. 2015:507151, 1–14. doi: 10.1155/2015/507151 26064103 PMC4443644

[ref76] Nadkarni V. D. Pervin A. Linhardt R. J. (1994). Directional immobilization of heparin onto beaded supports. Anal. Biochem. 222, 59–67. doi: 10.1006/abio.1994.1454, PMID: 7856872

[ref77] Nataraj B. H. Ali S. A. Behare P. V. Yadav H. (2020). Postbiotics-parabiotics: the new horizons in microbial biotherapy and functional foods. Microb. Cell Factories 19:168. doi: 10.1186/s12934-020-01426-w, PMID: 32819443 PMC7441679

[ref78] Nguyen S. G. Kim J. Guevarra R. B. Lee J.-H. Kim E. Kim S.-I.. (2016). Laminarin favorably modulates gut microbiota in mice fed a high-fat diet. Food Funct. 7, 4193–4201. doi: 10.1039/C6FO00929H, PMID: 27713958

[ref79] Oh W. K. Lee C. H. Lee M. S. Bae E. Y. Sohn C. B. Oh H.. (2005). Antidiabetic effects of extracts from *Psidium guajava*. J. Ethnopharmacol. 96, 411–415. doi: 10.1016/j.jep.2004.09.041, PMID: 15619559

[ref80] Paik W. Alonzo F. Knight K. L. (2019). Probiotic exopolysaccharide protects against *systemic Staphylococcus aureus* infection, inducing dual-functioning macrophages that restrict bacterial growth and limit inflammation. Infect. Immun. 87, e00791–e00718. doi: 10.1128/IAI.00791-18, PMID: 30396894 PMC6300633

[ref81] Park Y. Hong Y. N. Weyers A. Kim Y. S. Linhardt R. J. (2011). Polysaccharides and phytochemicals: a natural reservoir for the green synthesis of gold and silver nanoparticles. IET Nanobiotechnol. 5, 69–78. doi: 10.1049/iet-nbt.2010.0033, PMID: 21913788

[ref82] Patterson E. Ryan P. M. Cryan J. F. Dinan T. G. Ross R. P. Fitzgerald G. F.. (2016). Gut microbiota, obesity and diabetes. Postgrad. Med. J. 92, 286–300. doi: 10.1136/postgradmedj-2015-133285, PMID: 26912499

[ref83] Pawlaczyk I. Czerchawski L. Kuliczkowski W. Karolko B. Pilecki W. Witkiewicz W.. (2011). Anticoagulant and anti-platelet activity of polyphenolic-polysaccharide preparation isolated from the medicinal plant *Erigeron canadensis* L. Thromb. Res. 127, 328–340. doi: 10.1016/j.thromres.2010.11.031, PMID: 21172723

[ref84] Rajakovich L. J. Balskus E. P. (2019). Metabolic functions of the human gut microbiota: the role of metalloenzymes. Nat. Prod. Rep. 36, 593–625. doi: 10.1039/C8NP00074C, PMID: 30452039 PMC7771511

[ref85] Ru Y. Liu K. Kong X. Li X. Shi X. Chen H. (2020). Synthesis of selenylated polysaccharides from *Momordica charantia* L. and its hypoglycemic activity in streptozotocin-induced diabetic mice. Int. J. Biol. Macromol. 152, 295–304. doi: 10.1016/j.ijbiomac.2020.02.288, PMID: 32112833

[ref86] Schepetkin I. A. Quinn M. T. (2006). Botanical polysaccharides: macrophage immunomodulation and therapeutic potential. Int. Immunopharmacol. 6, 317–333. doi: 10.1016/j.intimp.2005.10.005, PMID: 16428067

[ref87] Shan X. Zhou J. Ma T. Chai Q. (2011). *Lycium barbarum* polysaccharides reduce exercise-induced oxidative stress. Int. J. Mol. Sci. 12, 1081–1088. doi: 10.3390/ijms12021081, PMID: 21541044 PMC3083691

[ref88] Shang Q. Jiang H. Cai C. Hao J. Li G. Yu G. (2018). Gut microbiota fermentation of marine polysaccharides and its effects on intestinal ecology: an overview. Carbohydr. Polym. 179, 173–185. doi: 10.1016/j.carbpol.2017.09.059, PMID: 29111040

[ref89] Shen X. Wang Z. Song X. Xu J. Jiang C. Zhao Y.. (2014). Transcriptomic profiling revealed an important role of cell wall remodeling and ethylene signaling pathway during salt acclimation in *Arabidopsis*. Plant Mol. Biol. 86, 303–317. doi: 10.1007/s11103-014-0230-9, PMID: 25092201

[ref90] Sinha V. R. Kumria R. (2001). Polysaccharides in colon-specific drug delivery. Int. J. Pharm. 224, 19–38. doi: 10.1016/S0378-5173(01)00720-7, PMID: 11472812

[ref91] Song S. K. Beck B. R. Kim D. Park J. Kim J. Kim H. D.. (2014). Prebiotics as immunostimulants in aquaculture: a review. Fish Shellfish Immunol. 40, 40–48. doi: 10.1016/j.fsi.2014.06.016, PMID: 24973515

[ref92] Song J. Chen M. Li Z. Zhang J. Hu H. Tong X.. (2019). Astragalus polysaccharide extends lifespan via mitigating endoplasmic reticulum stress in the silkworm, *Bombyx mori*. Aging Dis. 10, 1187–1198. doi: 10.14336/AD.2019.0515, PMID: 31788331 PMC6844597

[ref93] Souza R. O. S. Assreuy A. M. S. Madeira J. C. Chagas F. D. S. Parreiras L. A. Santos G. R. C.. (2015). Purified polysaccharides of *Geoffroea spinosa* barks have anticoagulant and antithrombotic activities devoid of hemorrhagic risks. Carbohydr. Polym. 124, 208–215. doi: 10.1016/j.carbpol.2015.01.069, PMID: 25839813

[ref94] Sun S.-S. Wang K. Ma K. Bao L. Liu H.-W. (2019). An insoluble polysaccharide from the sclerotium of *Poria cocos* improves hyperglycemia, hyperlipidemia and hepatic steatosis in *Ob/Ob* mice via modulation of gut microbiota. Chin. J. Nat. Med. 17, 3–14. doi: 10.1016/S1875-5364(19)30003-2, PMID: 30704621

[ref95] Surayot U. Wangtueai S. You S. Palanisamy S. Krusong W. Brennan C. S.. (2021). Extraction, structural characterisation, and immunomodulatory properties of edible *Amanita hemibapha* subspecies *Javanica* (corner and bas) mucilage polysaccharide as a potential of functional food. J Fungi (Basel) 7:683. doi: 10.3390/jof7090683, PMID: 34575721 PMC8468940

[ref96] Tan X. Zhou X. Chen H.-G. (2017). Structure-activity relationship of plant polysaccharides. Zhongguo Zhong Yao Za Zhi 42, 4104–4109. doi: 10.19540/j.cnki.cjcmm.20170928.016, PMID: 29271146

[ref97] Tomé L. C. Fernandes S. C. M. Perez D. S. Sadocco P. Silvestre A. J. D. Neto C. P.. (2013). The role of nanocellulose fibers, starch and chitosan on multipolysaccharide based films. Cellulose 20, 1807–1818. doi: 10.1007/s10570-013-9959-6

[ref98] Wang K. Niu M. Song D. Song X. Zhao J. Wu Y.. (2020a). Preparation, partial characterization and biological activity of exopolysaccharides produced from *Lactobacillus fermentum* S1. J. Biosci. Bioeng. 129, 206–214. doi: 10.1016/j.jbiosc.2019.07.009, PMID: 31471140

[ref99] Wang L.-Y. Wang Y. Xu D.-S. Ruan K.-F. Feng Y. Wang S. (2012). MDG-1, a polysaccharide from *Ophiopogon japonicus* exerts hypoglycemic effects through the PI3K/Akt pathway in a diabetic KKAy mouse model. J. Ethnopharmacol. 143, 347–354. doi: 10.1016/j.jep.2012.06.050, PMID: 22776833

[ref100] Wang Y. Wang X. Zhang K. Zhang X. Li S. Li Y.. (2020). Extraction kinetics, thermodynamics, rheological properties and anti-BVDV activity of the hot water assisted extraction of Glycyrrhiza polysaccharide. Food Funct. 11, 4067–4080. doi: 10.1039/d0fo00608d, PMID: 32329761

[ref101] Wang M. Xie Z. Li L. Chen Y. Li Y. Wang Y.. (2019). Supplementation with compound polysaccharides contributes to the development and metabolic activity of young rat intestinal microbiota. Food Funct. 10, 2658–2675. doi: 10.1039/c8fo02565g, PMID: 31025991

[ref102] Wang L. Xie Y. Yang W. Yang Z. Jiang S. Zhang C.. (2019a). Alfalfa polysaccharide prevents H2O2-induced oxidative damage in MEFs by activating MAPK/Nrf2 signaling pathways and suppressing NF-κB signaling pathways. Sci. Rep. 9:1782. doi: 10.1038/s41598-018-38466-7, PMID: 30742052 PMC6370797

[ref1001] Wang X. Wang X. Jiang, H. Cai C. Li G. Hao J.. (2018). Marine polysaccharides attenuate metabolic syndrome by fermentation products and altering gut microbiota: An overview. Carbohydr. Polym. 195, 601–612. doi: 10.1016/j.carbpol.2018.05.003 29805017

[ref103] Wang G. Yang X. Wang J. Zhong D. Zhang R. Zhang Y.. (2021). Walnut green husk polysaccharides prevent obesity, chronic inflammatory responses, nonalcoholic fatty liver disease and colonic tissue damage in high-fat diet fed rats. Int. J. Biol. Macromol. 182, 879–898. doi: 10.1016/j.ijbiomac.2021.04.047, PMID: 33857511

[ref104] Wang K. Yang X. Wu Z. Wang H. Li Q. Mei H.. (2020b). *Dendrobium officinale* polysaccharide protected CCl4-induced liver fibrosis through intestinal homeostasis and the LPS-TLR4-NF-κB signaling pathway. Front. Pharmacol. 11:240. doi: 10.3389/fphar.2020.00240, PMID: 32226380 PMC7080991

[ref105] Wang L. Zhang X. Niu Y. Ahmed A. F. Wang J. Kang W. (2019b). Anticoagulant activity of two novel polysaccharides from flowers of *Apocynum venetum* L. Int. J. Biol. Macromol. 124, 1230–1237. doi: 10.1016/j.ijbiomac.2018.12.015, PMID: 30521914

[ref106] Wei H. Shi Y. Yuan Z. Huang Z. Cai F. Zhu J.. (2021). Isolation, identification, and anti-inflammatory activity of polysaccharides of *Typha angustifolia*. Biomacromolecules 22, 2451–2459. doi: 10.1021/acs.biomac.1c00235, PMID: 34024108

[ref107] Witvrouw M. Este J. A. Mateu M. Q. Reymen D. Andrei G. Snoeck R.. (1994). Activity of a Sulfated polysaccharide extracted from the red seaweed *Aghardhiella Tenera* against human immunodeficiency virus and other enveloped viruses. Antivir. Chem. Chemother. 5, 297–303. doi: 10.1177/095632029400500503

[ref108] Wu J. Shi S. Wang H. Wang S. (2016). Mechanisms underlying the effect of polysaccharides in the treatment of type 2 diabetes: a review. Carbohydr. Polym. 144, 474–494. doi: 10.1016/j.carbpol.2016.02.040, PMID: 27083840

[ref109] Wu Y. Wan J. Choe U. Pham Q. Schoene N. W. He Q.. (2019). Interactions between food and gut microbiota: impact on human health. Annu. Rev. Food Sci. Technol. 10, 389–408. doi: 10.1146/annurev-food-032818-121303 30908952

[ref110] Wu J. Xu Y. Liu X. Chen M. Zhu B. Wang H.. (2020). Isolation and structural characterization of a non-competitive α-glucosidase inhibitory polysaccharide from the seeds of *Litchi chinensis Sonn*. Int. J. Biol. Macromol. 154, 1105–1115. doi: 10.1016/j.ijbiomac.2019.11.170, PMID: 31760014

[ref111] Wu S. Zhang X. Liu J. Song J. Yu P. Chen P.. (2019). Physicochemical characterization of *Sargassum fusiforme* fucoidan fractions and their antagonistic effect against P-selectin-mediated cell adhesion. Int. J. Biol. Macromol. 133, 656–662. doi: 10.1016/j.ijbiomac.2019.03.218, PMID: 30930270

[ref112] Xie J.-H. Jin M.-L. Morris G. A. Zha X.-Q. Chen H.-Q. Yi Y.. (2016). Advances on bioactive polysaccharides from medicinal plants. Crit. Rev. Food Sci. Nutr. 56, S60–S84. doi: 10.1080/10408398.2015.1069255 26463231

[ref113] Xu R. Aruhan Xiu L. Sheng S. Liang Y. Zhang H.. (2019). Exopolysaccharides from *Lactobacillus buchneri* TCP016 attenuate LPS- and d-GalN-induced liver injury by modulating the gut microbiota. J. Agric. Food Chem. 67, 11627–11637. doi: 10.1021/acs.jafc.9b04323, PMID: 31553177

[ref114] Xu X. Shan B. Liao C.-H. Xie J.-H. Wen P.-W. Shi J.-Y. (2015). Anti-diabetic properties of *Momordica charantia* L. polysaccharide in alloxan-induced diabetic mice. Int. J. Biol. Macromol. 81, 538–543. doi: 10.1016/j.ijbiomac.2015.08.049, PMID: 26318666

[ref115] Xu Y. Wu Y.-J. Sun P.-L. Zhang F.-M. Linhardt R. J. Zhang A.-Q. (2019). Chemically modified polysaccharides: synthesis, characterization, structure activity relationships of action. Int. J. Biol. Macromol. 132, 970–977. doi: 10.1016/j.ijbiomac.2019.03.213, PMID: 30965077

[ref116] Xu R. Ye H. Sun Y. Tu Y. Zeng X. (2012). Preparation, preliminary characterization, antioxidant, hepatoprotective and antitumor activities of polysaccharides from the flower of tea plant (*Camellia sinensis*). Food Chem. Toxicol. 50, 2473–2480. doi: 10.1016/j.fct.2011.10.047, PMID: 22033094

[ref117] Xue Y.-T. Li S. Liu W.-J. Xin M. Li H.-H. Yu G.-L.. (2018). The mechanisms of sulfated polysaccharide drug of propylene glycol alginate sodium sulfate (PSS) on bleeding side effect. Carbohydr. Polym. 194, 365–374. doi: 10.1016/j.carbpol.2018.04.048, PMID: 29801851

[ref118] Yang Y. Ji J. di L. Li J. Hu L. Qiao H.. (2020). Resource, chemical structure and activity of natural polysaccharides against alcoholic liver damages. Carbohydr. Polym. 241:116355. doi: 10.1016/j.carbpol.2020.116355, PMID: 32507196

[ref119] Yao Y. Yan L. Chen H. Wu N. Wang W. Wang D. (2020). *Cyclocarya paliurus* polysaccharides alleviate type 2 diabetic symptoms by modulating gut microbiota and short-chain fatty acids. Phytomedicine 77:153268. doi: 10.1016/j.phymed.2020.153268, PMID: 32663709

[ref120] Yoon S.-J. Yu M.-A. Pyun Y.-R. Hwang J.-K. Chu D.-C. Juneja L. R.. (2003). The nontoxic mushroom *Auricularia auricula* contains a polysaccharide with anticoagulant activity mediated by antithrombin. Thromb. Res. 112, 151–158. doi: 10.1016/j.thromres.2003.10.022, PMID: 14967412

[ref121] You L. Gao Q. Feng M. Yang B. Ren J. Gu L.. (2013). Structural characterisation of polysaccharides from *Tricholoma matsutake* and their antioxidant and antitumour activities. Food Chem. 138, 2242–2249. doi: 10.1016/j.foodchem.2012.11.140, PMID: 23497882

[ref122] Yu Y. Zhu H. Shen M. Yu Q. Chen Y. Xie J. (2021). Sulfation modification enhances the intestinal regulation of *Cyclocarya paliurus* polysaccharides in cyclophosphamide-treated mice via restoring intestinal mucosal barrier function and modulating gut microbiota. Food Funct. 12, 12278–12290. doi: 10.1039/D1FO03042F, PMID: 34821227

[ref123] Zeng P. Li J. Chen Y. Zhang L. (2019). The structures and biological functions of polysaccharides from traditional Chinese herbs. Prog. Mol. Biol. Transl. Sci. 163, 423–444. doi: 10.1016/bs.pmbts.2019.03.003, PMID: 31030757 PMC7102684

[ref124] Zhang L. Hao J. Zheng Y. Su R. Liao Y. Gong X.. (2018). Fucoidan protects dopaminergic neurons by enhancing the mitochondrial function in a rotenone-induced rat model of Parkinson’s disease. Aging Dis. 9, 590–604. doi: 10.14336/AD.2017.0831, PMID: 30090649 PMC6065300

[ref125] Zhang R. Yuan S. Ye J. Wang X. Zhang X. Shen J.. (2020). Polysaccharide from *Flammuliana velutipes* improves colitis via regulation of colonic microbial dysbiosis and inflammatory responses. Int. J. Biol. Macromol. 149, 1252–1261. doi: 10.1016/j.ijbiomac.2020.02.044, PMID: 32035958

[ref126] Zhang Z. Zhang L. Xu H. (2019). Effect of Astragalus polysaccharide in treatment of diabetes mellitus: a narrative review. J. Tradit. Chin. Med. 39, 133–138. doi: 10.19852/j.cnki.jtcm.2019.01.017 PMID: 32186034

[ref127] Zhang X. Zhao S. Song X. Jia J. Zhang Z. Zhou H.. (2018). Inhibition effect of glycyrrhiza polysaccharide (GCP) on tumor growth through regulation of the gut microbiota composition. J. Pharmacol. Sci. 137, 324–332. doi: 10.1016/j.jphs.2018.03.006, PMID: 30150145

[ref128] Zhao R. Ji Y. Chen X. Hu Q. Zhao L. (2021). Polysaccharide from *Flammulina velutipes* attenuates markers of metabolic syndrome by modulating the gut microbiota and lipid metabolism in high fat diet-fed mice. Food Funct. 12, 6964–6980. doi: 10.1039/D1FO00534K, PMID: 34137411

[ref129] Zhao R. Ji Y. Chen X. Su A. Ma G. Chen G.. (2020). Effects of a β-type glycosidic polysaccharide from *Flammulina velutipes* on anti-inflammation and gut microbiota modulation in colitis mice. Food Funct. 11, 4259–4274. doi: 10.1039/C9FO03017D, PMID: 32356528

[ref130] Zhou R. He D. Xie J. Zhou Q. Zeng H. Li H.. (2021). The synergistic effects of polysaccharides and ginsenosides from American ginseng (*Panax quinquefolius* L.) ameliorating cyclophosphamide-induced intestinal immune disorders and gut barrier dysfunctions based on microbiome-metabolomics analysis. Front. Immunol. 12:665901. doi: 10.3389/fimmu.2021.665901, PMID: 33968068 PMC8100215

[ref131] Zhou N. Long H. Wang C. Yu L. Zhao M. Liu X. (2020). Research progress on the biological activities of selenium polysaccharides. Food Funct. 11, 4834–4852. doi: 10.1039/C9FO02026H, PMID: 32478773

